# Compliance to perioperative anticoagulation protocols in elderly patients undergoing elective orthopedic procedures: a retrospective observational cohort study on 548 patients

**DOI:** 10.1186/s13037-023-00357-w

**Published:** 2023-04-20

**Authors:** Lizzie Munk, Tom van Essen, Casper van der Hoeven, Peter A. Nolte, Matthijs L. Becker

**Affiliations:** 1 Pharmacy Foundation of Haarlem Hospitals, Haarlem, the Netherlands; 2 Department of Clinical Pharmacy, Spaarne Gasthuis Hospital, Hoofddorp, the Netherlands; 3 Department of Clinical Pharmacy, Rode Kruis Hospital, Beverwijk, the Netherlands; 4 Department of Orthopedics, Noordwest Ziekenhuisgroep, Alkmaar, the Netherlands; 5 Department of Anaesthesiology, Spaarne Gasthuis Hospital, Hoofddorp, the Netherlands; 6 Department of Orthopedics, Spaarne Gasthuis Hospital, Hoofddorp, the Netherlands; 7 Department of Oral Cell Biology, Academic Center for Dentistry Amsterdam, Vrije Universiteit Amsterdam, Amsterdam Movement Sciences, Gustav Mahlerlaan 3004, 1081 LA Amsterdam, the Netherlands

**Keywords:** Orthopedic surgery, Elective surgical procedures, Anticoagulants, Guideline compliance, Thromboembolism

## Abstract

**Background:**

Compliance with perioperative anticoagulation guidelines is essential to minimize bleeding and thromboembolic risks in patients undergoing surgery. Compared to vitamin-K antagonists (VKAs), perioperative management of direct oral anticoagulants (DOACs) contains fewer steps. Therefore, we hypothesized that noncompliance with guidelines in VKA users is higher than in DOAC users. The primary aim of our study was to investigate the difference in noncompliance to perioperative anticoagulant management guidelines between elderly patients using VKAs versus those using DOACs. The secondary aim was to determine the difference in occurrence of conflicting information communicated to the patients and the difference in incidence of coagulation-related adverse events.

**Methods:**

This retrospective non-controlled observational cohort study examined elderly patients undergoing elective orthopedic surgery in a teaching hospital in the Netherlands. All patients undergoing elective orthopedic surgery between 1 May 2016 and 1 January 2020, aged 70 years and over, using VKAs or DOACs were selected. Nonelective surgeries were excluded. The primary outcome was the noncompliance to perioperative anticoagulant management guidelines. Secondary outcomes were missing or conflicting information on anticoagulation management communicated to the patient and coagulation-related adverse events. For continuous data, the unpaired T-test was used and for categorical data, the chi-square test.

**Results:**

In patients using VKAs, noncompliance to one of the steps of perioperative anticoagulation management was 81%, compared to 55% in patients using DOACs (*p* < 0.001). In most cases, VKAs or DOACs were interrupted for longer than recommended. In 13% of patients using a VKA with perioperative bridging, bridging was not conducted as recommended in the guidelines. In 13% of patients using a DOAC, a low-molecular-weight heparin (LMWH) was prescribed while a DOAC had already been restarted postoperatively. VKA users received conflicting information about perioperative anticoagulation management more often than DOAC users (33% versus 20%; *p* < 0.001). No difference was seen in postoperative coagulation-related complications.

**Conclusion:**

Guidelines compliance in DOAC users is higher than in VKA users. Clinical decision support to help in selecting the right interruption interval in DOAC users, simplified standardized perioperative management, good coordination of instructions given to patients, and familiarity with updated guidelines are important in reducing noncompliance.

**Supplementary Information:**

The online version contains supplementary material available at 10.1186/s13037-023-00357-w.

## Background

In patients treated with anticoagulants who are in need of elective surgery, the right perioperative anticoagulation management should be executed to minimize the risk of both bleeding and thromboembolic complications [1–5]. This is especially the case for elderly patients who often have multiple comorbidities and are at an increased risk for bleeding complications [6].

**Fig. 1:**
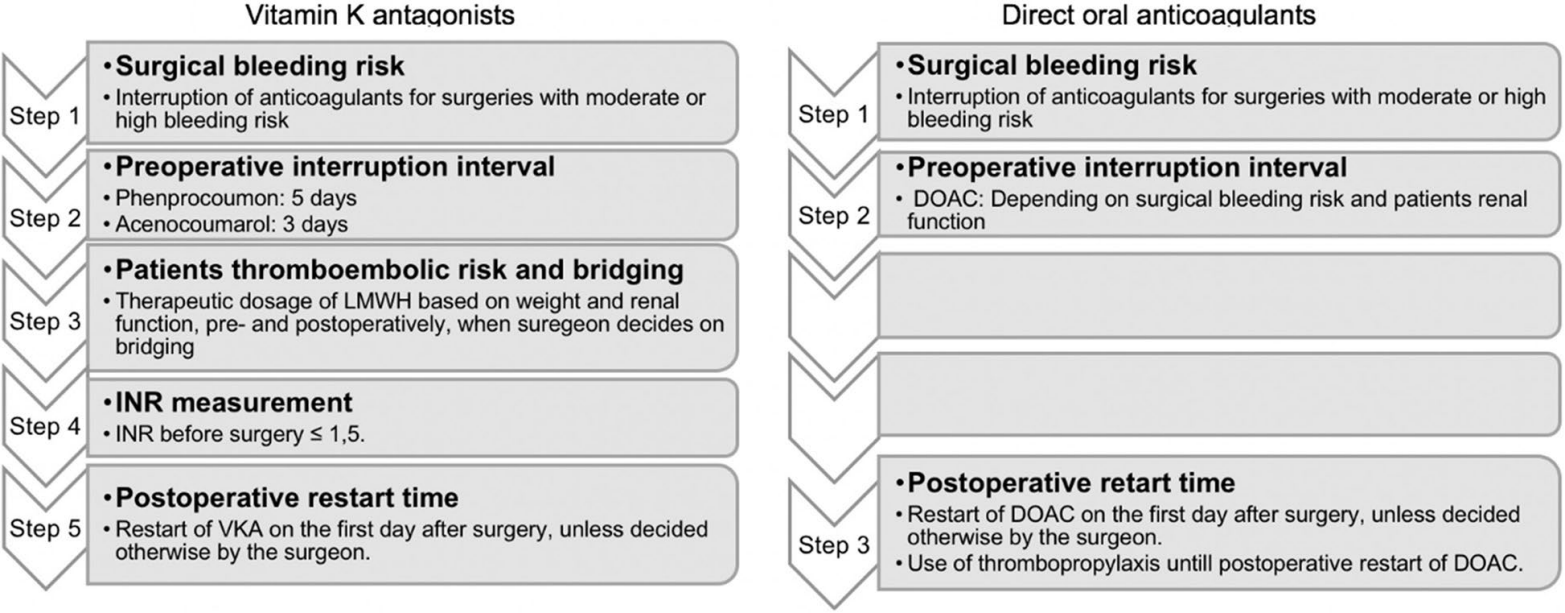
Perioperative anticoagulation management steps for Vitamin K antagonists and DOACs. DOAC: Direct oral anticoagulants, INR: International Normalized Ratio, LMWH: Low-molecular-weight heparin, VKA: Vitamin K antagonist

Perioperative anticoagulation management is a high-risk and challenging process; multiple healthcare professionals are involved, and the process contains multiple steps (Fig. 1). For a long time, the periprocedural management of direct oral anticoagulants (DOACs) was based on expert reviews instead of clinical data, which led to various guidelines and unwanted variation in local protocols [7–12]. These guidelines suggested periprocedural management for patients usings DOACs based on the DOAC used, renal function, and procedural bleeding risk [13, 14]. With the periprocedural management suggested in these guidelines, there was a low risk of thrombotic and bleeding complications, with no significant difference compared to the interruption of vitamin K antagonists (VKAs) [2]. In the PAUSE cohort study, a simple standardized perioperative management approach was studied [5]. DOACs were omitted for 1 day before a low-bleeding-risk procedure and for 2 days before a high-bleeding-risk-procedure, except for patients using dabigatran with an eGFR less than 50 ml/min. This approach resulted in low rates of perioperative bleeding and thromboembolism.

At the time of this study, neither the American College of Chest Physicians (CHEST) guidelines on perioperative management of antithrombotic therapy nor the American Academy of Orthopaedic Surgeons guidelines on perioperative management of chronic anticoagulation in orthopedic surgery mentioned interruption intervals for DOAC therapy [15, 16]. In the American College of Cardiology 2017 periprocedural management of anticoagulation guidelines, DOAC intervals for dabigatran and the other DOACs were given depending on procedural bleeding risk and renal function [9]. In the Netherlands, two guidelines were published concerning perioperative anticoagulation management in patients undergoing elective surgery that were applicable during the study period [17, 18]. In these guidelines, the interruption interval for DOACs depended on the DOAC used, renal function, and the procedural bleeding risk; these intervals deviated from the ACC 2017 guidelines (Supplementary Table 1). Previous studies have shown that the compliance to the perioperative anticoagulation guidelines is poor [19–22]. However, these studies focused on patients using VKAs, and less is known about the guidelines compliance in patients using DOACs.

In the past decade, the number of DOAC users has increased, and the number of VKA users has decreased [23, 24]. Because perioperative anticoagulation management in DOAC users contains fewer steps (Fig. 1), we hypothesize that guidelines noncompliance in VKA users is higher than in DOAC users. The primary aim of this study is to investigate the difference in guidelines noncompliance of perioperative anticoagulation management in elderly VKA and DOAC users undergoing elective orthopedic surgery. Regarding anticoagulation management prior to hospital admission, differentiation is made between noncompliance by the physician and noncompliance by the patient. Secondary aims are to investigate whether information regarding anticoagulation management is recorded in the medical file, the difference in occurrence of conflicting information on perioperative anticoagulation management communicated to the patients as described in medical records, incidence of coagulation-related adverse events, and perioperative blood loss and blood transfusions.

## Methods

### Design and setting

This study is a retrospective noncontrolled observational cohort study of elderly patients using anticoagulants and undergoing elective surgery at the department of orthopedic surgery in the Spaarne Gasthuis Hospital, a teaching hospital in Hoofddorp, the Netherlands. The local perioperative anticoagulation management protocol of the Spaarne Gasthuis Hospital is based on the national guidelines of the Knowledge Institute of Medical Specialists (KIMS). At the Spaarne Gasthuis Hospital, patients undergoing elective surgery visit an anesthesiologist for preoperative screening and a pharmacy technician for medication reconciliation before surgery. The orthopedic surgeon is responsible for selecting and executing the appropriate perioperative anticoagulation management.

### Study population

All patients aged 70 years or older, using a VKA or DOAC and undergoing elective orthopedic surgery between 1 May 2016 and 1 January 2020, were included. Whether patients used a VKA or DOAC was based on prescriptions at the time of medication reconciliation. An exclusion criterion was nonelective surgery because the process of perioperative anticoagulation management is different from that for elective surgeries. For patients undergoing multiple surgeries during the study period, each surgery was included separately in the analyses.

### Data

Baseline characteristics such as sex, age, laboratory results, ASA classification, type of orthopedic surgery, and the individual complication registration were extracted from the electronic hospital information system Epic (Madison, WI, USA), using Crystal Reports (Walldorf, Germany). Drug use was extracted, and VKA or DOAC use as well as perioperative use of platelet aggregation inhibitors and tranexamic acid were verified by reviewing medical records. Comorbidities were assessed by reviewing preoperative screening reports. Bleeding risk of surgeries was based on the local perioperative anticoagulation management protocol of the hospital and classified by one researcher (PN). Blood transfusions, blood loss during surgery, whether a tourniquet was used during surgery to create a bloodless operative field, and whether a Bellovac drain was placed during surgery were assessed by reviewing operative reports.

### Outcome parameters

**Fig. 2:**
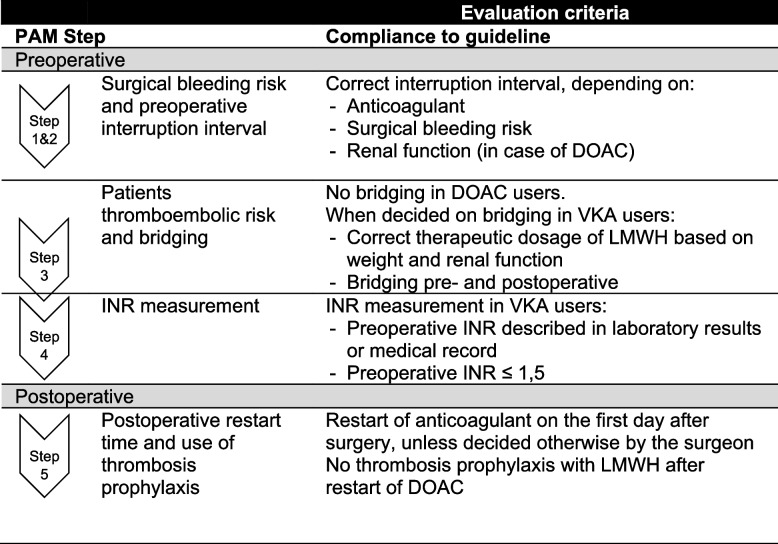
Evaluation criteria of compliance to perioperative anticoagulant management protocol. PAM: perioperative anticoagulation management DOAC: Direct oral anticoagulants, INR: International Normalized Ratio, LMWH: Low-molecular-weight heparin, VKA: Vitamin K antagonist

The primary outcome of this study—noncompliance to perioperative anticoagulation management guidelines—was defined as noncompliance to one or more steps of perioperative anticoagulation management (Fig. 2). Noncompliance was assessed by reviewing medical records and characteristics including laboratory results (International Normalized Ratio [INR], renal function [estimated Glomerular Filtration Rate]) and patient weight. When medical records did not contain the exact timing of interruption of anticoagulants as executed by the patient, the assumption was made that patients followed the last stated advice on the timing of interruption in their medical record. Acenocoumarol should be discontinued 3 days before surgery, phenprocoumon 5 days, and the discontinuation of DOACs as stated in the local protocol (see Supplementary Table 1).

Nonreporting of anticoagulation management was assessed by reviewing the medical records. If none of the healthcare records mentioned the interruption interval—either as decided by the physician or as executed by the patient—this was regarded as nonreporting. Conflicting information from different healthcare professionals communicated to the patient on perioperative anticoagulation management in medical records was assessed by reviewing medical records. All notes made by healthcare professionals from the moment the decision was made to operate to the operation itself were reviewed by LM. All information on the anticoagulation management in these records was compared. If any information mismatched the number of days of interruption or the date on which interruption had to be started, the information was noted as confusing. Coagulation-related adverse events were defined as the need to administer vitamin K, the presence of coagulation factors and/or blood transfusions perioperatively until 6 weeks after surgery, the occurrence of unexpected blood loss of more than 500 ml during surgery, and the presence of coagulation-related complications such as thromboembolic and bleeding complications until 6 weeks after surgery. The administering of vitamin K or coagulation factors was assessed for VKA users and based on prescriptions before surgery. During preoperative screening, all surgeons answered a standard question about expected blood loss during surgery. The answer to this question—combined with the amount of blood loss during surgery—was used to determine whether a blood loss of more than 500 ml was expected. Coagulation-related complications were based on complication registration and verified by reviewing medical records.

### Statistics

Statistical analyses were performed using IBM SPSS software, version 24. For continuous data, such as age and blood loss during surgery, an unpaired T-test was used to determine a difference between VKA and DOAC users. For all other categorical outcomes, a chi-square test was used. For all tests, a *p*-value < 0.05 was considered statistically significant.

### Ethics, funding, and potential conflicts of interest

The study was conducted according to the principles of the Declaration of Helsinki (October 2013 version) and in accordance with the General Data Protection Regulation. The research protocol was reviewed and approved by the local Institutional Review Board of the Spaarne Gasthuis Hospital. No approval from the ethics committee was required because this was a non-interventional study. The study has not received any funding, and none of the authors has any conflicts of interest to declare.

## Results

### Study population

**Fig. 3:**
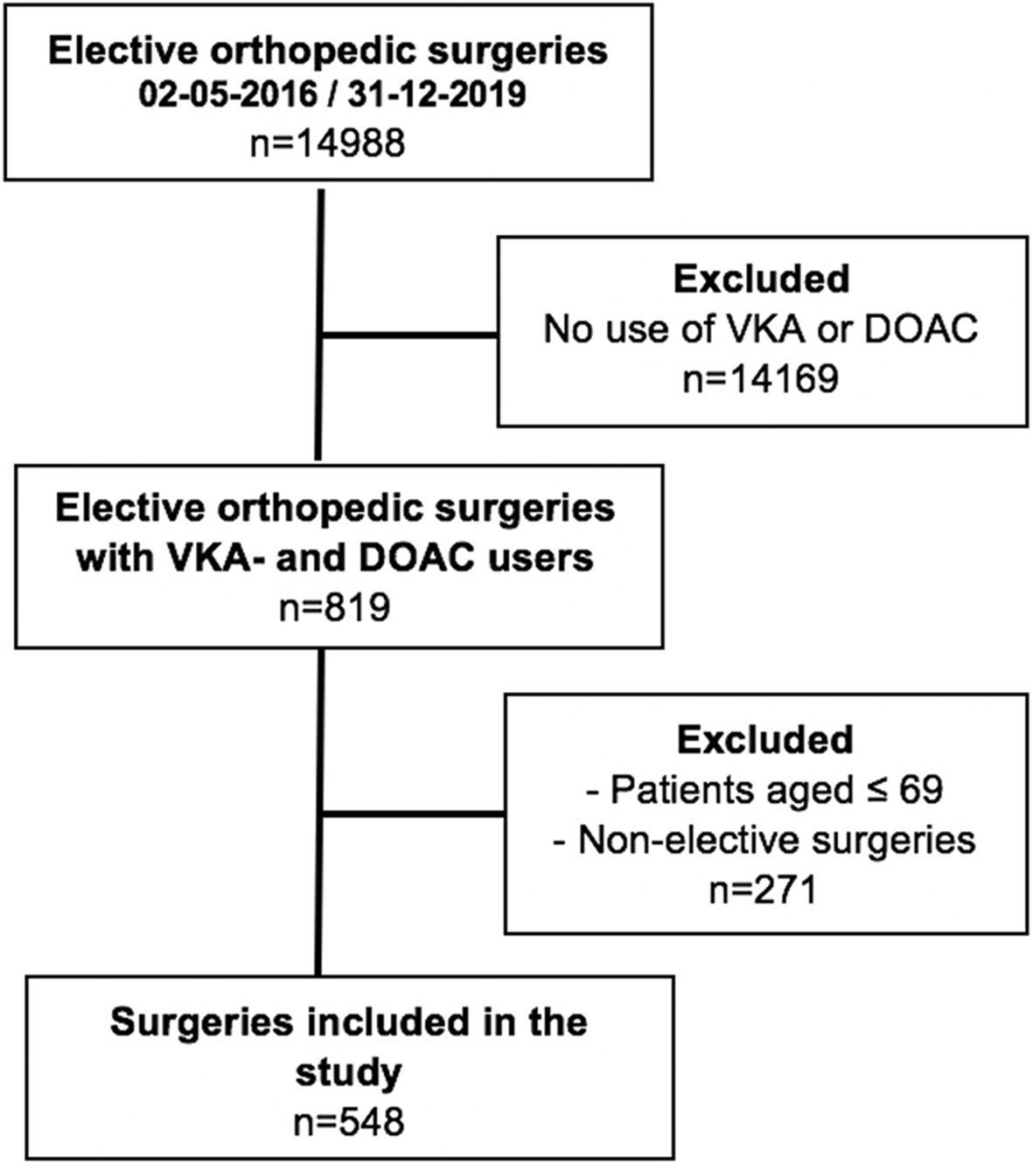
Patient inclusion flowchart. DOAC: Direct oral anticoagulants, VKA: Vitamin K antagonist

We included 548 elective orthopedic surgeries in our analyses. In 319 surgeries (58%), the patient used a VKA and in 229 surgeries a DOAC (42%; Fig. 3). During the study period, there was a decrease in the number of patients using VKAs and an increase in the number of patients using DOACs (Supplemental Figure 1). The mean age of patients was 78.2 years in VKA users and 77.5 years in DOAC users. Of all included surgeries, 30% were total knee arthroplasties (27% in VKA users and 35% in DOAC users), and 36% were total hip arthroplasties (36% in VKA users and 37% in DOAC users) (Table 1).

**Table 1:** Baseline characteristics of included surgeries

	**VKA users** ***n* = 319**		**DOAC users** ***n* = 229**		***P*-value**
Sex, (%)					0.7
Male	46		48		
Female	54		52		
Mean age (SD)	78,2	(5,2)	77,5	(4,9)	0.2
VKA users (%)					
Acenocoumarol	53				
Phenprocoumon	47				
DOAC users (%)					
Dabigatran			33		
Rivaroxaban			31		
Apixaban			28		
Edoxaban			8		
TAI users (%)	1		1		0.7
Administrating tranexamic acid (%)	30		39		0.02
Comorbidities (%)					
Coronary heart disease	19		23		0.3
CVA/TIA	20		19		0.7
Decompensatio cordis	20		14		0.1
Diabetes mellitus	3		6		0.7
Valvular heart disease	23		10		< 0.001
Hypertension	67		63		0.4
Peripheral arterial disease	4		8		0.1
Heart rhythm disorder	84		91		0.02
Thrombosis	17		12		0.1
Clotting disorder	1		0		0.1
Renal impairment (%)	13		9		0.2
eGFR 30—50	11		8		
eGFR < 30	2		1		
Surgical bleeding risk (%)					0.3
Low	0		0		
Intermediate	17		14		
High	83		86		
ASA score (%)					0.3
II	50		50		
III	48		49		
IV	2		1		
Tourniquet use (%)	22		26		0.3
Bellovac drain (%)	15		13		0.4

*DOAC* Direct oral anticoagulants, *eGFR* estimated glomerular filtration rate in ml/min, *TAI* thrombocyte aggregation inhibitors, *VKA* Vitamin K antagonist

### Primary outcome: noncompliance to the perioperative anticoagulation management guidelines

Noncompliance to one of the steps of perioperative anticoagulation management occurred more often in surgeries of VKA users than of DOAC users (81% versus 55%; *p* < 0.001). Noncompliance to the perioperative anticoagulation management guidelines is displayed in Table 2, and details on the steps in which noncompliance occurred are presented in Table 3. Noncompliance was highest in step 2 of the perioperative anticoagulation management, in which the timing of discontinuation of the anticoagulants before the surgery is determined. The timing of interruption varied from 2 to 14 days before surgery for patients using a VKA and from 1 to 11 days before surgery for patients using a DOAC. In 217 of 319 VKA users (68%) and 76 of 229 DOAC users (33%), the interruption interval was longer than recommended in the guidelines (*p* < 0.001). In most of these cases, the interruption interval was executed by the patient as advised by the physician (VKA 78%; DOAC 72%) and the noncompliance was because of the physician. In the other cases (VKA 22%; DOAC 28%), the noncompliance was because the patient did not follow the physician’s advice. In 11 VKA users (3%) and 15 DOAC users (7%), the interruption interval was shorter than recommended. In 18% of these VKA users and 80% of these DOAC users, the interruption interval was executed by the patient as advised by the physician, while in 82% of VKA users and 20% of DOAC users the noncompliance was because the patient did not follow the physician’s advice.

**Table 2:** Non-compliance to perioperative anticoagulation management guideline

	**VKA users** ***N* = 319**		**DOAC users** ***N* = 229**		***P*-value**
Non-compliance to PAM guideline, total (%)	257	(81%)	125	(55%)	< 0.001
Non-compliance to PAM guideline, preoperative (%)	253	(79%)	102	(45%)	< 0.001
Non-compliance to PAM guideline, postoperative (%)	11	(3%)	34	(15%)	< 0.001

*PAM* Perioperative anticoagulation management

**Table 3:**
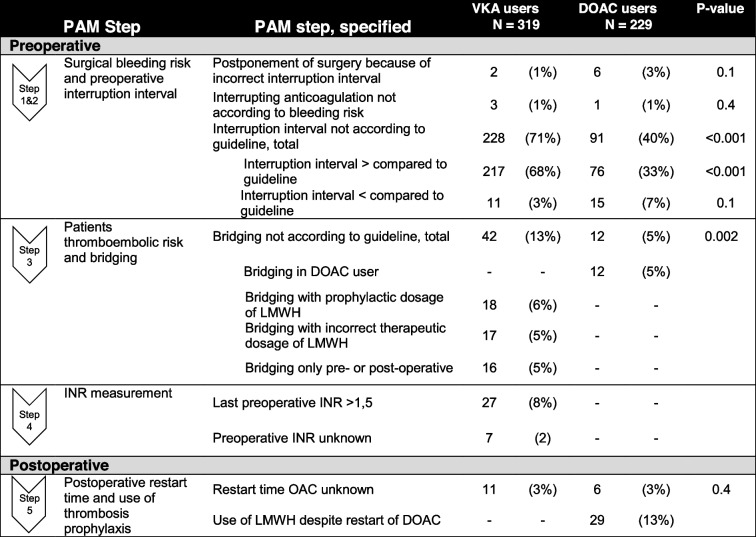
Non-compliance to perioperative anticoagulation management guideline, specified per step

*PAM* perioperative anticoagulation management, *DOAC* Direct oral anticoagulants, *INR* International Normalized Ratio, *LMWH* Low-molecular-weight heparin, *VKA* Vitamin K antagonist

Two surgeries in VKA users were cancelled because the INR was above 2 despite the correct timing of interruption and administering of vitamin K. Six surgeries in DOAC users were cancelled because DOAC use was not interrupted before surgery. Two of the six patients received incorrect information, and four of the six patients did not correctly execute the information they received.

Bridging with low-molecular-weight heparin (LMWH) during the interruption of VKA or DOAC use occurred in 51 of 319 surgeries with VKA users (16%) and in 12 of 229 of surgeries with DOAC users (5%). In 42 of 51 (82%) of surgeries with bridging in VKA users, bridging was not performed according to guidelines. In DOAC users, bridging with LMWHs is never indicated according to the guidelines.

### Secondary outcomes: conflicting information, blood loss during surgery, and adverse events

Secondary outcomes are displayed in Table 4. Of the 548 patients, 152 (28%) received conflicting information about the timing of interruption. In surgeries of VKA users, blood loss during surgery was higher and administering of blood transfusions occurred more often compared to surgeries with DOAC users. There were no differences in the occurrence of coagulation-related adverse events between the two groups.

**Table 4:** Secondary outcomes

	**VKA users** ***N* = 319**		**DOAC users** ***N* = 229**		***P*-value**
Preoperative (%)					
Administration of vitamin K	28	(9%)	-	-	
Administration of coagulations factors	1	(0%)	-	-	
Non-reporting of anticoagulation management as decided by pbysician	15	(5%)	4	(2%)	0.06
Non-reporting of exact timing of interruption as executed by patient	174	(55%)	92	(40%)	< 0.001
Conflicting information about perioperative anticoagulant management in medical records	106	(33%)	46	(20%)	< 0.001
Perioperative (%)					
Mean blood loss during surgery (SD)	252	(227)	210	(145)	0.041
> 500 ml unexpected blood loss during surgery	12	(4%)	2	(1%)	0.106
Postoperative untill six weeks after surgery					
Coagulation-related complications, total	37	(12%)	22	(10%)	0.10
Tromboembolic complications	2	(1%)	6	(3%)	0.06
Bleeding complications	37	(12%)	20	(9%)	0.30
Clinical relevant non-major bleeding	29	(9%)	18	(8%)	0.61
Major bleeding	8	(3%)	2	(1%)	0.16
Administration of blood transfusion	24	(8%)	6	(2%)	0.013

*DOAC* Direct oral anticoagulants, *VKA* Vitamin K antagonist, *SD* standard deviation

## Discussion

In patients who use VKAs and have elective orthopedic surgery, the risk of perioperative anticoagulation management noncompliance was higher compared to patients who use a DOAC and have elective orthopedic surgery. This is in line with our expectations because perioperative anticoagulation management in DOAC users contains fewer steps than perioperative anticoagulation management in VKA users. Nevertheless, the noncompliance remains high in DOAC users, with 55% of patients having noncompliance with at least one step of perioperative anticoagulation management. Moreover, 28% of patients received conflicting information from various healthcare providers on the timing of interruption.

In VKA users, noncompliance was most prevalent in the preoperative steps of perioperative anticoagulation management. In 68% of these patients, the interruption interval was longer than recommended by the guidelines, and in most patients this was advised by the physician. The longer interruption interval was prompted by the experience that the advised interruption interval for VKAs was too short and resulted in too many cancellations of surgeries. Therefore, an interruption interval of 5 days for acenocoumarol and 7 days for phenprocoumon was used. This interruption interval may increase the risk of thromboembolic events in these patients. However, the risk of these events in patients with atrial fibrillation, which is the most prevalent indication of anticoagulants in our population, is low. In the BRIDGE trial, the risk of thromboembolism was 0.4% in patients with atrial fibrillation who were not bridged with an LMWH, and this risk was not significantly different compared to patients with atrial fibrillation who were bridged with an LMWH [1]. In the BRIDGE trial, patients were using warfarin, and warfarin therapy was stopped 5 days before surgery; the average CHADS2 score in this trial was 2.3. Although not the subject of this trial, it is to be expected that the thromboembolic risk is not substantially increased if VKA therapy is stopped several days earlier. This is especially the case in high-risk patients because anticoagulation therapy is bridged with an LMWH, reducing the thromboembolic risk. In 3% of patients, the interruption interval was shorter than recommended by the guidelines with the potential of a too-high INR before surgery, in most cases because of noncompliance by the patient. Because INR is measured preoperatively, patients with too-high INR levels before surgery are intercepted and treated with vitamin K as needed. Bridging was performed incorrectly in 13% of VKA users, mostly because of incorrect doses prescribed.

In DOAC users, the percentage of patients with a timing of interruption not according to the guidelines was 40% (91 of 229 patients) compared to 71% in VKA users. We noticed that superseded versions of guidelines on the perioperative management of DOACs were still used in the study period, such as the 2012 guidelines ‘guided introduction on new oral anticoagulants’ [25]. In DOAC users, renal function was incorporated in the judgment regardless of whether the interruption interval was in line with the guidelines as presented in Supplementary Table 1. The percentage of patients with an interruption interval shorter than recommended in the guidelines was 7% in DOAC users compared to 3% in VKA users. Because anticoagulation levels are not measured before surgery in DOAC users, unlike VKA users in whom INR is measured, too short of an interruption interval in DOAC users exposes them to a potential increased bleeding risk during surgery. In most cases, the patient executed the interruption interval as instructed by the physician, and the noncompliance was because of the physician. Moreover, we found that in 5% of patients, DOAC therapy was bridged with LMWH therapy—although not recommended in the guidelines—and in 13% of patients, LMWH was used postoperatively together with DOAC therapy erroneously. However, the overall noncompliance is lower in DOAC users than in VKA users; we found that DOAC users are exposed to a higher bleeding risk because of a too-short interruption interval and erroneous use of DOAC and LMWH combined. Contrary to this, we see in the secondary end points that perioperative blood loss during surgery and the administration of blood transfusions were lower in patients using DOACs, suggesting that DOAC use might be safer than VKA use. However, because the numbers were low and differences small, no hard conclusions should be drawn. Implementation of a simplified standardized perioperative management approach, as suggested in the PAUSE study, may result in higher guidelines compliance by the physician [5]. This approach has been implemented in the CHEST 2022 guidelines on perioperative management of antithrombotic therapy [11].

Previous studies on noncompliance with perioperative anticoagulation management guidelines have shown poor compliance, similar to the results found in our study [19–22]. Moesker et al. described guidelines compliance of 40–81%, depending on the step of the perioperative anticoagulation management [21, 22]. Contrary to our study, they found that the incidence of guidelines noncompliance was highest in the postoperative steps when VKA therapy was reinitiated. An explanation for this could be that we assessed whether the operator made a conscious decision concerning the day of reinitiating VKA therapy after surgery. In the study by Moesker et al., guidelines compliance in 48 DOAC users was analyzed, and the interruption interval was more frequently too long than too short, as compared to the guidelines [20]. For 3 of 34 patients (9%) in whom the interruption interval was analyzable, the interruption interval was too short. This percentage is comparable to the 7% found in our study.

Our study has several shortcomings and limitations. First, we retrospectively analyzed patient dossiers and depended on what was reported in them. The moment of stopping anticoagulation therapy before surgery was not reported for all patients, and assumptions had to be made. If a decision deviated from the guidelines, in most cases no reason was mentioned—and we do not know whether the noncompliance was intentional or erroneous. Moreover, we do not know whether patients complied with the instructions by their physician or reported their use correctly. Second, there were differences in baseline characteristics between the VKA users and DOAC users, which might have influenced the results. Because the percentage of DOAC users increased over time, a change in perioperative anticoagulation management compliance over time might have influenced the difference in guidelines compliance between VKA and DOAC users. However, we have not seen any trend in compliance over time. Third, in the patients using DOACs, more patients preoperatively received tranexaminic acid. During the study period, the guidelines concerning the administration of tranexaminic acid were changed, and the administration was not recommended in the new guidelines. Nevertheless, preoperative administration of tranexaminic acid was higher in patients using DOACs. It is possible that patients using VKAs had more comorbidities, such as valvular heart disease, and therefore more frequently had a contra-indication for the administration of tranexaminic acid. This might have influenced the risk of bleeding complications. Fourth, we analyzed peri- and postoperative bleeding complications as a secondary outcome. However, complications depend on many factors that were not part of the scope of this study, and we therefore cannot draw conclusions on this.

Our study shows that, although guidelines compliance is higher in the group of patients using DOACs, compliance with these guidelines is still limited. Moreover, we identified that one in four patients was given conflicting information about the timing of interruption. Decision support given by the hospital information system could be used to optimize perioperative anticoagulation management guidelines compliance. During the study period, several changes were made to the hospital information system, including mandatory questions about the anticoagulants used and the advised interruption period in the process of ordering a surgery. The orthopedic surgeon must complete these questions and therefore must be aware of whether a patient uses anticoagulants and make an explicit decision on the interruption interval. Because the guidelines recommendations are based on drug use, renal function, and the bleeding risk of the surgery, an algorithm could be developed that gives advice on the interruption interval. In addition, good coordination among healthcare providers is important because many providers are involved in perioperative anticoagulation management.

## Conclusion

With the increasing use of DOACs in elderly patients undergoing orthopedic surgery, anticoagulation management has become easier. Guidelines compliance in DOAC users is higher compared to VKA users. The noncompliance with the highest risk for bleeding complications is a too-short interruption interval before surgery in patients using DOACs. Decision support in the hospital information system can help in selecting the right interruption interval, based on renal function and the bleeding risk of the surgery. Another strategy could be to implement a simplified standardized perioperative management system that is independent of the renal function for all DOACs except dabigatran. All healthcare providers involved in perioperative anticoagulation should coordinate and communicate the anticoagulation management for each patient with one another to make sure that the patient is not given conflicting information. The providers should be familiar with the most recent guidelines; we noticed that noncompliance was frequently because superseded guidelines were being followed.

## Supplementary Information

**Additional file 1: Supplementary table 1.** Interruption time of DOAC as stated in local protocol. **Supplementary Figure 1.** Use of VKA and DOAC over the years in the study population.

## Data Availability

The datasets used and/or analysed during the current study are available from the corresponding author on reasonable request.

## Abbreviations

DOAC: Direct oral anticoagulants
INR: International Normalized Ratio
KIMS: Knowledge Institute of Medical Specialists
LMWH: Low-molecular-weight heparin
VKA: Vitamin K antagonists

## References

[1] Douketis JD, Spyropoulos AC, Kaatz S, Becker RC, Caprini JA, Dunn AS, Garcia DA, Jacobson A, Jaffer AK, Kong DF, Schulman S, Turpie AG, Hasselblad V, Ortel TL, BRIDGE Investigators (2015). Perioperative bridging anticoagulation in patients with atrial fibrillation. N Engl J Med.

[2] Shaw JR, Woodfine JD, Douketis J, Schulman S, Carrier M (2018). Perioperative interruption of direct oral anticoagulants in patients with atrial fibrillation: a systematic review and meta-analysis. Res Pract Thromb Haemost.

[3] Ferrandis R, Llau JV, Sanz JF, Cassinello CM, González-Larrocha O, Matoses SM, Suárez V, Guilabert P, Torres LM, Fernández-Bañuls E, García-Cebrián C, Sierra P, Barquero M, Montón N, Martínez-Escribano C, Llácer M, Gómez-Luque A, Martín J, Hidalgo F, Yanes G, Rodríguez R, Castaño B, Duro E, Tapia B, Pérez A, Villanueva AM, Álvarez JC, Sabaté S, RA-ACOD investigators (2020). Periprocedural Direct Oral Anticoagulant Management: The RA-ACOD Prospective, Multicenter Real-World Registry. TH Open.

[4] Shah S, Nayfeh T, Hasan B, Spyropoulos AC, Douketis JD, Murad MH (2022). Perioperative management of vitamin K antagonists and direct oral anticoagulants; a systematic review and meta-analysis. Chest.

[5] Douketis JD, Spyropoulos AC, Duncan J, Carrier M, Le Gal G, Tafur AJ, Vanassche T, Verhamme P, Shivakumar S, Gross PL, Lee AYY, Yeo E, Solymoss S, Kassis J, Le Templier G, Kowalski S, Blostein M, Shah V, MacKay E, Wu C, Clark NP, Bates SM, Spencer FA, Arnaoutoglou E, Coppens M, Arnold DM, Caprini JA, Li N, Moffat KA, Syed S, Schulman S (2019). Perioperative management of patients with atrial fibrillation receiving a direct oral anticoagulant. JAMA Intern Med.

[6] Palareti G, Cosmi B (2009). Bleeding with anticoagulation therapy - who is at risk, and how best to identify such patients. Thromb Haemost.

[7] Raval AN, Cigarroa JE, Chung MK, Diaz-Sandoval LJ, Diercks D, Piccini JP, Jung HS, Washam JB, Welch BG, Zazulia AR, Collins SP, American Heart Association Clinical Pharmacology Subcommittee of the Acute Cardiac Care and General Cardiology Committee of the Council on Clinical Cardiology, Council on Cardiovascular Disease in the Young, Council on Quality of Care and Outcomes Research (2017). Management of patients on non-vitamin K antagonist oral anticoagulants in the acute care and periprocedural setting: a scientific statement from the American Heart Association. Circulation.

[8] Steffel J, Verhamme P, Potpara TS, Albaladejo P, Antz M, Desteghe L, Haeusler KG, Oldgren J, Reinecke H, Roldan-Schilling V, Rowell N, Sinnaeve P, Collins R, Camm AJ, Heidbüchel H, ESC Scientific Document Group (2018). The 2018 European Heart Rhythm Association Practical Guide on the use of non-vitamin K antagonist oral anticoagulants in patients with atrial fibrillation. Eur Heart J.

[9] Doherty JU, Gluckman TJ, Hucker WJ, Januzzi JL, Ortel TL, Saxonhouse SJ, Spinler SA (2017). 2017 ACC Expert Consensus Decision Pathway for Periprocedural Management of Anticoagulation in Patients With Nonvalvular Atrial Fibrillation: A Report of the American College of Cardiology Clinical Expert Consensus Document Task Force. J Am Coll Cardiol.

[10] Squizzato A, Poli D, Barcellona D, Ciampa A, Grandone E, Manotti C, Moia M, Toschi V, Tosetto A, Testa S, Scientific Reviewer Committee (2022). Management of DOAC in Patients Undergoing Planned Surgery or Invasive Procedure: Italian Federation of Centers for the Diagnosis of Thrombotic Disorders and the Surveillance of the Antithrombotic Therapies (FCSA) Position Paper. Thromb Haemost.

[11] Douketis JD, Spyropoulos AC, Murad MH, Arcelus JI, Dager WE, Dunn AS, Fargo RA, Levy JH, Samama CM, Shah SH, Sherwood MW, Tafur AJ, Tang LV, Moores LK (2022). Perioperative management of antithrombotic therapy: an American College of Chest Physicians Clinical Practice Guideline. Chest.

[12] Mitrovic D, van Elp M, Veeger N, Lameijer H, Meijer K, van Roon E (2023). Protocols for perioperative management of direct oral anticoagulants in hospitals: opportunities for improvement. Curr Med Res Opin.

[13] Spyropoulos AC, Al-Badri A, Sherwood MW, Douketis JD (2016). Periprocedural management of patients receiving a vitamin K antagonist or a direct oral anticoagulant requiring an elective procedure or surgery. J Thromb Haemost.

[14] Spyropoulos AC, Douketis JD (2012). How I treat anticoagulated patients undergoing an elective procedure or surgery. Blood.

[15] Douketis JD, Spyropoulos AC, Spencer FA, Mayr M, Jaffer AK, Eckman MH, Dunn AS, Kunz R (2012). Perioperative management of antithrombotic therapy: Antithrombotic Therapy and Prevention of Thrombosis, 9^th^ ed: American College of Chest Physicians Evidence-Based Clinical Practice Guidelines. Chest.

[16] Thakur NA, Czerwein JK, Butera JN, Palumbo MA (2010). Perioperative management of chronic anticoagulation in orthopaedic surgery. J Am Acad Orthop Surg.

[17] Knowledge Institute of Medical Specialists (KIMS). 2014, National Standard Chain Care Anticoagulation [Landelijke Standaard Ketenzorg Antistolling]. Available from: https://www.knmp.nl/media/833. Accessed: 7 June 2022.

[18] Knowledge Institute of Medical Specialists (KIMS). 2016. Antithrombotic Policy. [Antitrombotisch beleid]. Available from: https://www.trombosestichting.nl/wp-content/uploads/2019/11/Antitrombotisch-Beleid-Preventie-VTE-bij-ingrepen.pdf. Accessed: 7 June 2022.

[19] Eijgenraam P, ten Cate H, ten Cate-Hoek AJ (2014). Practice of bridging anticoagulation: guideline adherence and risk factors for bleeding. Neth J Med.

[20] Moesker MJ, Damen NL, de Groot JF, de Bruijne MC, Wagner C. Utrecht/Amsterdam: NIVEL/EMGO+, 2017. Anticoagulant care in Dutch hospitals. Evaluation of thrombosis prophylaxis and perioperative anticoagulation policy compared to current guidelines. [Antistollingszorg Nederlandse ziekenhuizen. Evaluatie van tromboseprofylaxe en perioperatief antistollingsbeleid in vergelijking met geldende richtlijnen]. Available from: https://www.nivel.nl/nl/publicatie/antistollingszorg-nederlandse-ziekenhuizen-evaluatie-van-tromboseprofylaxe-en. Accessed: 6 June 2022.

[21] Moesker MJ, de Groot JF, Damen NL, Huisman MV, de Bruijne MC, Wagner C (2019). How reliable is perioperative anticoagulant management? Determining guideline compliance and practice variation by a retrospective patient record review. BMJ Open.

[22] Moesker MJ, de Groot JF, Damen NL, Bijsterveld NR, Twisk JWR, Huisman MV, de Bruijne MC, Wagner C (2019). Guideline compliance for bridging anticoagulation use in vitamin-K antagonist patients; practice variation and factors associated with non-compliance. Thromb J.

[23] Dutch Foundation for Pharmaceutical Statistics (SFK). DOAC continues to gain ground at VKA [DOAC blijft terrein winnen op VKA]. Pharmaceutisch Weekblad. 2019;154(37):9.

[24] Lippi G, Mattiuzzi C, Cervellin G, Favaloro EJ (2017). Direct oral anticoagulants: analysis of worldwide use and popularity using Google Trends. Ann Transl Med.

[25] Order of Medical Specialists (OMS). 2012, guideline guided introduction new oral anticoagulants [Leidraad begeleide introductie nieuwe orale antistollingsmiddelen]. Available from: https://www.nvkc.nl/apps/actueel/Documents/Leidraad_NOAC.pdf.

