# Supplementary material for: Compliance to perioperative anticoagulation protocols in elderly patients undergoing elective orthopedic procedures: a retrospective observational cohort study on 548 patients
Source: Patient Saf Surg. 2023 Apr 20;17:9. doi: 10.1186/s13037-023-00357-w (PMC10116649; doi:10.1186/s13037-023-00357-w)
Supplement: Supplementary file 1 — Additional file 1: Supplementary table 1. Interruption time of DOAC as stated in local protocol. Supplementary Figure 1. Use of VKA and DOAC over the years in the study population. [file 13037_2023_357_MOESM1_ESM.docx]

Supplementary data

Supplementary table 1: Interruption time of DOAC as stated in local protocol

| DOAC | *Renal function* | *Interruption time – surgery with*  *intermediate bleeding risk* | *Interruption time – surgery with high bleeding risk* |
| --- | --- | --- | --- |
| Dabigatran | eGFR>80  eGFR 50-80  eGFR 30-50  eGFR <30 | 24 hours  36 hours  48 hours  contraindicated | 48 hours  72 hours  96 hours  contraindicated |
| Rivaroxaban | eGFR>80  eGFR 50-80  eGFR 30-50  eGFR <30 | 24 hours  24 hours  24 hours  36 hours | 48 hours  48 hours  48 hours  48 hours |
| Apixaban | eGFR>80  eGFR 50-80  eGFR 30-50  eGFR <30 | 24 hours  24 hours  24 hours  36 hours | 48 hours  48 hours  48 hours  48 hours |
| Edoxaban | eGFR>80  eGFR 50-80  eGFR 30-50  eGFR <30 | 24 hours  Minimum of 24 hours  Minimum of 24 hours  Minimum of 36 hours | 48 hours  Minimum of 48 hours  Minimum of 48 hours  Minimum of 48 hours |

DOAC: Direct oral anticoagulants, eGFR: estimated glomerular filtration rate in ml/min.

Supplementary figure 1: Use of VKA and DOAC over the years in the study population
